# Development of a one-pot synthesis of rGO in water by optimizing Tour’s method parameters

**DOI:** 10.1038/s41598-024-73606-2

**Published:** 2024-09-27

**Authors:** Andrea Rossi, Eugenio Alladio, Damjana Drobne, Vasile-Dan Hodoroaba, Kerstin Jurkschat, Veno Kononenko, Loay Akmal Madbouly, Paul Mrkwitschka, Sara Novak, Jörg Radnik, Špela Saje, Rosangela Santalucia, Fabrizio Sordello, Francesco Pellegrino

**Affiliations:** 1 Department of Chemistry and NIS Centre, University of Torino, Via Giuria 7, Torino, 10125 Italy; 2 Department of Biology, Biotechnical Faculty, University of Ljubljana, Večna Pot 111, Ljubljana, SI-1000 Slovenia; 3 Federal Institute for Materials Research and Testing (BAM), Unter den Eichen 44-46, 12203 Berlin, Germany; 4 Department of Materials, Oxford University, Begbroke Science Park, Begbroke Hill, Yarnton, Oxford, OX5 1PF UK

**Keywords:** Graphene, 2D-materials, Tour’s method, RGO, One-pot synthesis, Chemistry, Materials science, Nanoscience and technology

## Abstract

Since its first synthesis in 2004, graphene has been widely studied and several different synthesis methods has been developed. Solvent exfoliation of graphite and the reduction of graphene oxide previously obtained through graphite oxidation are the most employed. In this work, we exploited synthesis conditions of a method usually employed for obtaining graphene oxide (the Tour’s method) for directly obtaining a very poorly oxidised material with characteristics like reduced graphene oxide. For the first time, a one-pot synthesis of reduced graphene oxide (rGO) is reported avoiding the use of a post-synthesis chemical or thermal reduction of the graphene oxide that requires further reagents, heat and time.

**Supplementary information:**

The online version contains supplementary material available at 10.1038/s41598-024-73606-2.

## Introduction

In the ever-evolving landscape of materials science, one discovery has captured the imagination of researchers, engineers, and enthusiasts alike: graphene. This two-dimensional arrangement of carbon atoms, in a hexagonal lattice, has emerged as a powerhouse in the realm of nanotechnology, promising groundbreaking applications across various industries^1,2^. Since its isolation in 2004 by Geim and Novoselov at the University of Manchester, graphene has sparked a revolution, opening up a plethora of possibilities that extend far beyond conventional materials^2,3^. From its unparalleled strength and conductivity to its transparency and flexibility, its unique attributes have positioned graphene as a transformative force in fields ranging from electronics and energy storage to medical devices and beyond^4–6^.

While the isolation of graphene by mechanical exfoliation, also known as the “Scotch tape method”, marked the initial breakthrough, various synthesis methods have since been developed to produce graphene on larger scale^7,8^. Given some difficulties in obtaining high quality graphene, one of the most popular alternative is the synthesis of the reduced graphene oxide (rGO). This material derives from the reduction of the functional groups present at the surface of graphene oxide^9,10^. The rGO has characteristics similar to the ones of the graphene, even if it still presents some heteroatoms and a certain defectivity in the structure. The reduction is usually conducted using chemicals like hydrazine, able to chemically reduce the oxygen containing functional groups^11,12^. However, hydrazine has a several problems due to its toxicity, flammability and explosiveness. Therefore, in the last few years, new approaches for the reduction of graphene oxide to rGO were developed, like the use of other chemicals with a safer profile (ascorbic acid) or the use of high temperatures^12–15^. The main drawback of these methods, as in the case of the hydrazine, is that a second step is always required for reduce the graphene oxide once obtained.

In this work, we overcome the need of a second reductive step by properly choosing the synthetic parameters of the Tour’s method, one of the most employed methods for the synthesis of graphene oxide. The one-pot rGO synthesis here developed provides a significant sustainability benefit, eliminating the need for a further chemical or thermal reduction.

## Results and discussion

In the synthesis, 100 mL solution of H_2_SO_4_/H_3_PO_4_ 50:50 was slowly added to 3 g of graphite in 2-liter three-necked round bottom flask under stirring. The suspension was kept 30 min in ice-bath (T < 5 °C) to avoid excessive heating. This initial step is fundamental for reducing the attractive forces among most of the graphite layers, facilitating the exfoliation. After 30 min, the ice bath was removed, and the graphite suspension in concentrated acid was vigorously stirred for an additional 4 h and 30 min.

**Fig. 1:**
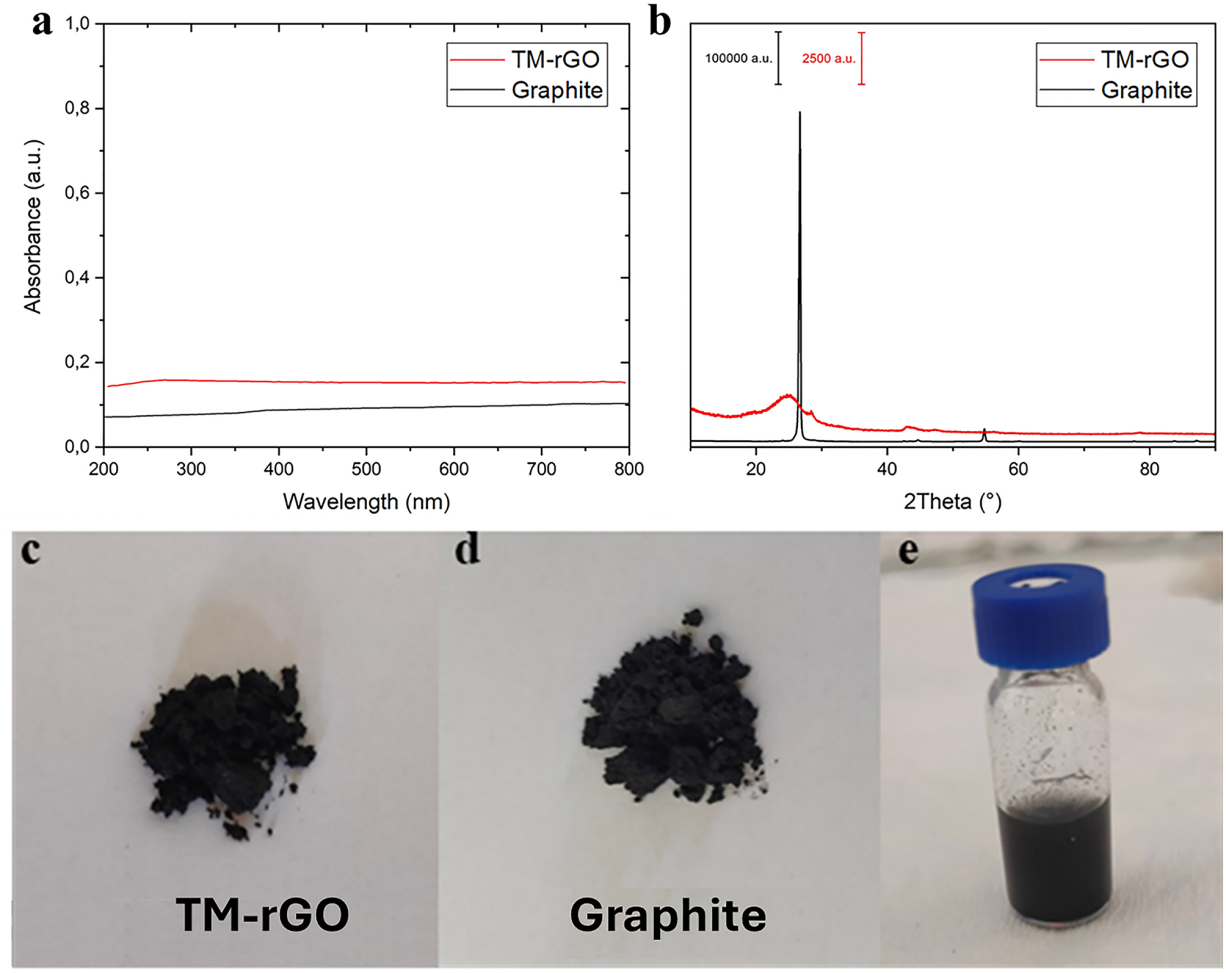
UV-Vis characterization at 20 mg L^−1^(**a**) and XRD pattern of the original graphite and the TM-rGO synthetised (**b**); pictures of the rGO (**c**), graphite powders (**d**) and rGO suspension in water at 1 g L^−1^ (**e**).

The total intercalation process lasted 5 h. After that, 6 g of KMnO_4_ were gradually added to the graphite suspension. This addition was done while the suspension was kept in ice-bath for keeping the temperature below 20 °C. After 30 min of stirring, the suspension was heated until 75 °C and stirred continuously for 96 h. Finally, the suspension was cooled down with ice-bath and 400 ml of water were slowly added. The obtained suspension was maintained under vigorous stirring for 24 h at room temperature. Finally, the reaction was stopped by adding H_2_O_2_ while keeping it in ice-bath. After the synthesis, the material was transferred into centrifuge tubes and subjected to a series of centrifugation cycles, in which the supernatant was discarded, and the precipitated re-suspended in 1 M HCl and then in ultrapure water. Following the purification process through centrifugation, the material was transferred into a dialysis membrane and dialyzed against ultrapure water for 15 days, with the external water being replaced at least twice daily. After the purification process, a portion of the material, suspended in ultrapure water, was transferred to a bottle and stored in the dark, while another portion was freeze-dried to obtain a dry powder for further characterization. During the entire process, the material was never sonicated for inducing exfoliation. The final yield of the reaction is more than 80%, with the greatest losses due to the cleaning procedure. Therefore the reaction can be considered almost quantitative. The synthesis was replicated to evaluate its reproducibility, proving to be reproducible at the limit of the experimental error. Results on chemical composition of the replicates are reported in SI. The synthetised material (named TM-rGO in the following) was systematically characterized with several techniques. First, UV-Vis of the suspension and the XRD of the powders were carried out (Fig. 1).

From the UV-Vis (Fig. 1a) it is possible to see that the absorption spectrum is almost flat along all the wavelength range considered, not so different from the one obtained for the graphite employed as precursor. A very small absorption peak centred at 265–270 nm in the spectrum of TM-rGO appears, that is typical of reduced graphene oxide^16^. This is the first proof of the direct synthesis of reduced graphene oxide rather than graphene oxide which, instead, is reported to have a double absorption at 230 and 300 nm^17,18^. Moreover, XRD characterisation (Fig. 1b) highlights the strong effect of the treatment on the diffraction pattern. The very intense and sharp peak of the graphite centred at 26.7° was completely changed into a small and broad peak centred at nearly 25°^16^. This pattern can be compared to the one of the rGO rather than that of graphene oxide reported in literature^16,17^. The great reduction of the peak intensity, the shift at lower angles, and the peak broadening are three symptoms of an effective exfoliation of the original material and a low oxidation level. Moreover, the peak at nearly 42° can be attributed at the (100) reflection peak typical of rGO. The peak centred at 28,6° is already present in the original graphite as highlighted in SI (Figure S1) and it is probably due to impurities The XRD data also explained the UV-Vis spectra obtained. In Fig. 1c and d we displayed the photographs of the powders studied. As clearly visible, the black colour of the material TM-rGO indicates a reduced degree of oxidation, while the typical colour for graphene oxide is brownish. In Fig. 1e the suspension of the material TM-rGO is showed, which again displays black colour without brown shades.

**Fig. 2:**
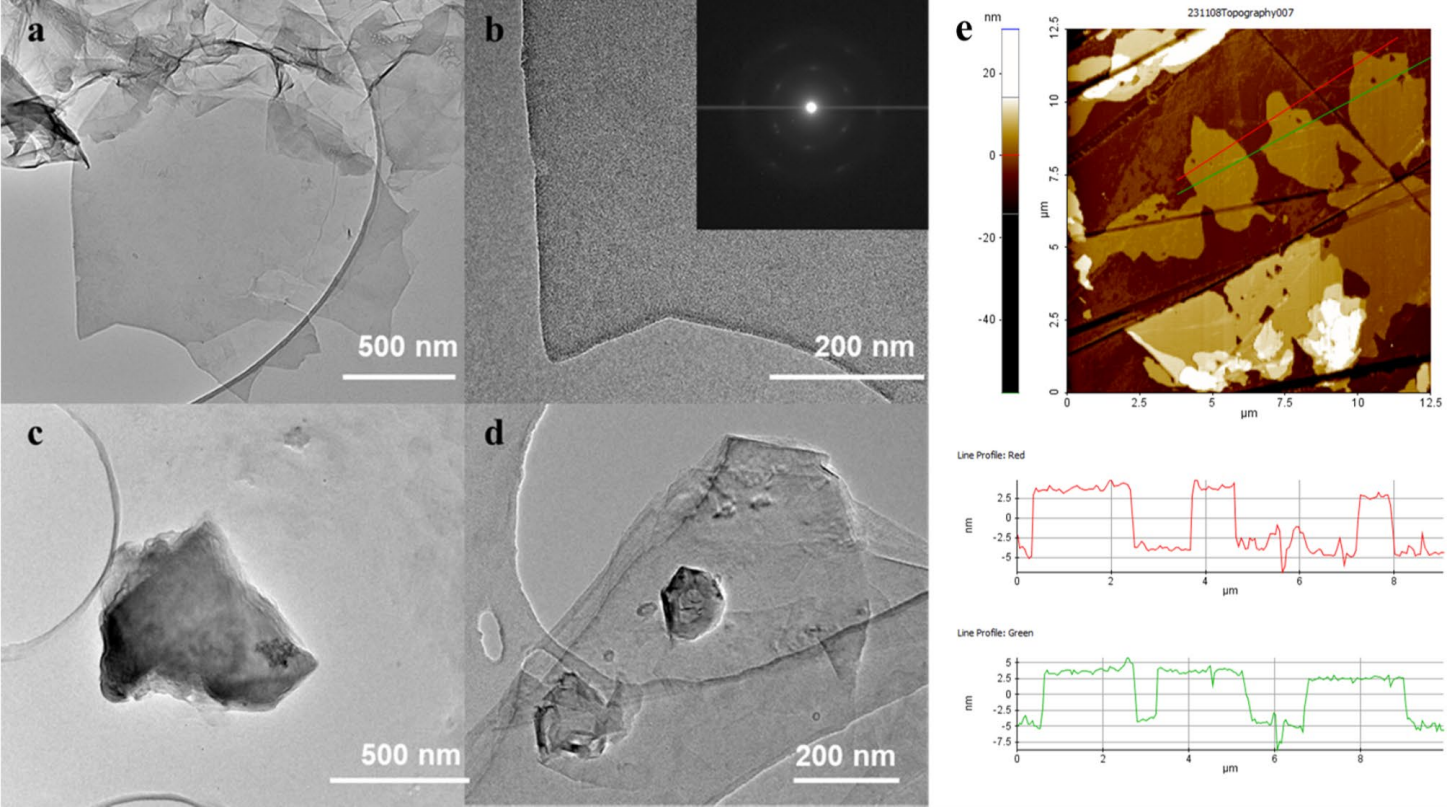
TEM micrographs obtained for the material TM-rGO (**a** and **b**) and for the graphite used as precursor (**c** and **d**); AFM topography of the material TM-rGO (**e**).

In Fig. 2 TEM micrographs of the synthetized material and the graphite precursor are reported. TM-rGO retained the jagged edges already present in the starting material, however, after the treatment the different stacked planes observable in the case of graphite (Fig. 2c and d) are now monolayers (curled up or not) clearly visible both in Fig. 2a and in Fig. 2b. Atomic Force Microscopy (AFM) was employed for the measurement of the thickness of the graphene flakes obtained after the exfoliation of graphite. For this, a diluted (0.5 g/L) water suspension of the material was deposited by drop-casting on a silicon wafer. As highlighted in Fig. 2e, the thickness of the graphene flakes is nearly 7–8 nm, indicating that the material is not constituted only by monolayer (which typically have a thickness of ∼0.8–1.5 nm)^19–21^, but often the presence of multilayers from 6 to 9 occurs.

**Fig. 3:**
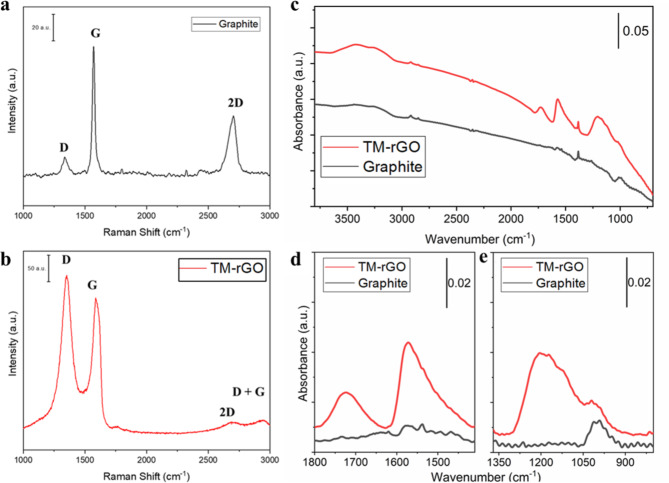
Left: Raman analysis of the original graphite (**a**) and of the TM-rGO (**b**). Right: (**c**) FT-IR spectra of TM-rGO sample (in red) and graphite (in dark grey). *IR feature due to the mis-compensation of atmospheric CO_2_ in the instrument when recording reference and sample spectra. † IR feature due to KNO_3_ impurity present in KBr powder. Enlarged views in the 1800 –1410 cm^−1^ (**d**) and 1370 –800 cm^−1^ (**e**) spectral regions of IR spectra reported in panel A after baseline correction (to reduce the effect of scattering).

In Fig. 3 we report the Raman analysis of the graphite and of the TM-rGO synthetised. After the treatment, the ratio I_D_/I_G_ largely increased from 0.10 for the graphite to 1.12 for the TM-rGO. The TM-rGO is therefore more defective, as underlined also by the presence of the D + G peak (2945 cm^−1^)^22,23^. The G peak of the graphite is shifted at higher wavenumber (from 1574 cm^−1^ to 1590 cm^−1^) while the 2D peak is shifted from 2708 cm^−1^ to 2692 cm^−1^.^24,25^ This is consistent with the exfoliation that occurred^26^. Additionally, the I_2D_/I_G_ ratio for TM-rGO is less than 1, indicating that the material is not primarily composed of monolayer graphene, as previously observed through AFM^23^.

To explore the chemical and structural changes occurring during the synthesis, the IR spectra of TM-rGO sample and graphite were examined in the range 3800 –700 cm^−1^ (Fig. 3c). It is expected that the IR spectrum of graphite to be flat due to the intrinsic nature of the ν(C = C) vibrational modes within conjugated double bonds in graphitic sp^2^ domains. These modes usually produce very weak signals in the IR spectrum because they are not linked to a change in dipole moment^27^.

However, in Fig. 3c several IR features are observed. Specifically, the vibrational modes of adsorbed water molecules in the 3600 –3200 cm^−1^ range (νO–H) and at 1620 cm^−1^ (δH_2_O), along with the narrow peak at 1384 cm^−1^, which can be attributed to the presence of KNO_3_ impurity, are associated with KBr powder used in the preparation of the IR pellets. Additionally, very weak bands originating from the vibrational modes of νC–H (3100 –2800 cm^−1^), νC = C (1600 –1420 cm^−1^), and νC–C/δC–H (1050 –900 cm^−1^), indicative of the skeletal vibration and C–H surface groups situated at the periphery of the sp^2^ clusters in the unoxidized graphitic network, are also observed.

Upon treatment of pristine graphite, some oxygen-containing functional groups, such as –COOH and C = O located at the sheet edge, as well as –OH and epoxy C–O on the basal planes of the GO sheet, can be generated. In Fig. 3c, there is an observable increase in the intensity of the O–H stretching band, attributed to the formation of OH groups and a slightly higher concentration of adsorbed H_2_O molecules. In Fig. 3d and e, the main changes in TM-rGO following graphite treatment can be observed in detail. Two distinct regions have been examined and interpreted separately^27–29^. The first region from 1800 cm^−1^ to 1410 cm^−1^ (Fig. 3d) highlights the stretching mode of C = O group, centered at 1724 cm^−1^, suggesting the formation of carbonyl/carboxyl groups at the edges of the graphene flakes. The spectral feature centered around 1570 cm^−1^ aligns with the C = C stretching mode, indicating the retention of residual sp^2^ character after the treatment of the graphitic network. However, the broadness of this peak suggests the presence of additional overlapping peaks, such as the C–OH deformation mode, whose absorption falls within 1500 –1350 cm^−1^ range. The second region of interest is between 1370 cm^−1^ and 800 cm^−1^ (Fig. 3e). The intense and very broad absorption in the 1300–900 cm^−1^ region is due to the overlapping of numerous absorption bands, making detailed assignment challenging. In this range, IR features associated with C–O bonds (1230–1210 cm^−1^), epoxy species (1120 –1110 cm^−1^), alkoxy or epoxy moieties (1080 –1040 cm^−1^), in-plane δ(C–H) modes and collective skeletal C–C vibrational modes (1300 –1000 cm^−1^), are detected. Additionally, signals attributed to sulfate groups (formed upon oxidation of the graphitic network) are also present within this region. The FTIR analysis confirmed the presence of some oxygen-containing functional groups in the TM-rGO structure that are completely absent in the graphite. However, their presence is limited compared to typical graphene oxide materials produced through the Tour’s method. Moreover, the weak signal given by the water molecules absorbed on the surface of the material suggests a good degree of hydrophobicity. These characteristics are coherent with structure of rGOrather than graphene oxide. Typically, the intensity of the ν(C = O) band provides an indication of the degree of graphene oxide oxidation. In the material TM-rGO, the ν(C = O) band intensity appears much less pronounced compared to the C = C stretching band, consistent with the material being closer to rGO. However, due to overlapping absorption bands and varying absorption coefficients, interpreting the intensity of the ν(C = O) band relative to the C = C stretching band should be done with caution. A more accurate assessment of the carbon content in different forms will be provided below by the XPS spectra, which offer greater specificity and clarity.

**Fig. 4:**
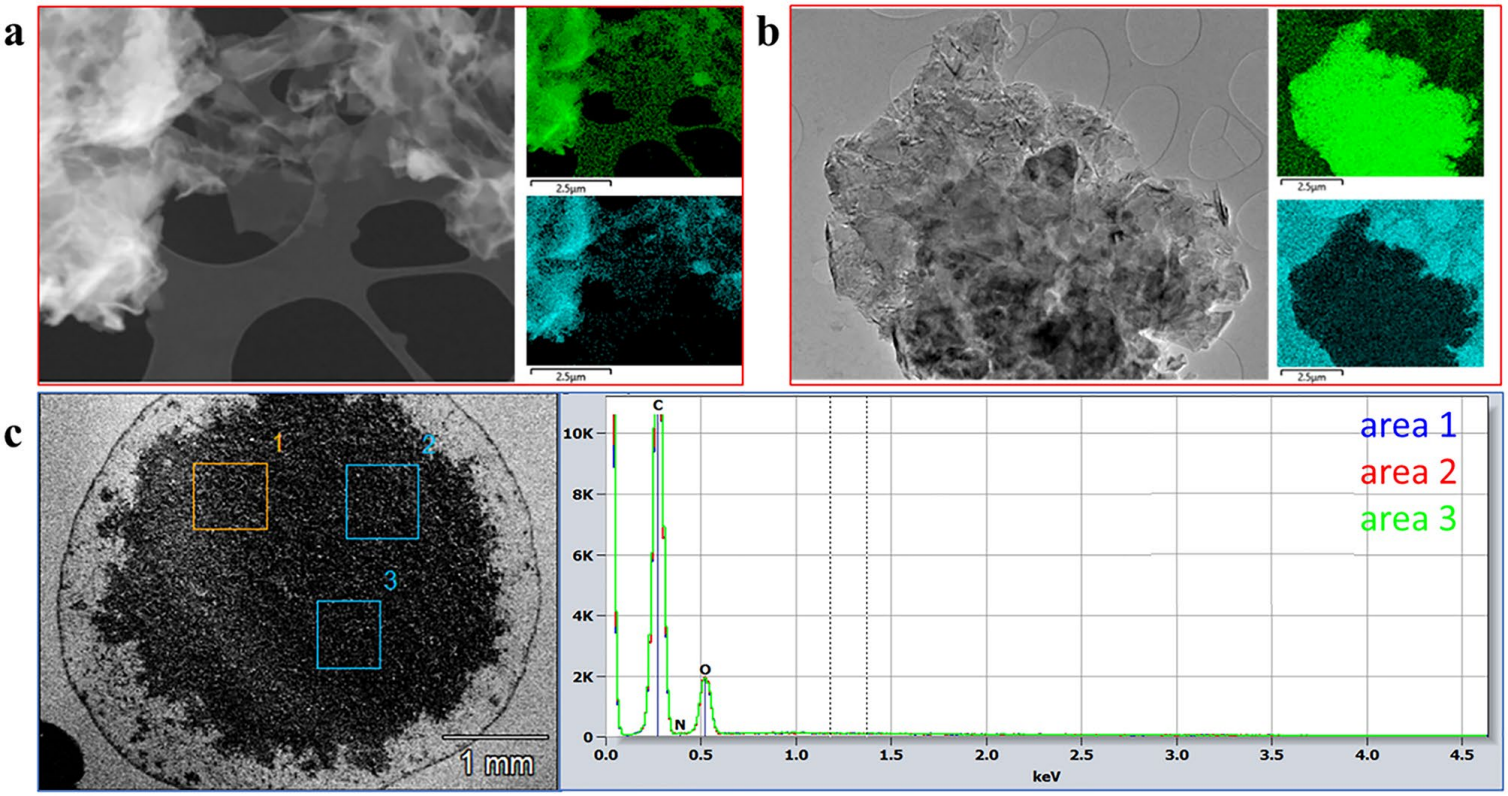
TEM/EDX map analysis of the material TM-rGO (**a**) and of the graphite (**b**). In green the C distribution and in cyan the O distribution; SEM/EDX results of the TM-rGO (**c**).

The chemical composition of the materials was also assessed by EDX (TEM/EDX and SEM/EDX) and XPS analysis. The O/C atom-% ratio obtained from TEM/EDX for the material TM-rGO (Fig. 4a) is nearly 0.2, a value similar to those reported for rGO from literature and that clearly confirms the presence of small amount of oxygen^30,31^. Typical graphene oxide values of EDX are close to 0.4 or more^32,33^. In Fig. 4b a representative TEM/EDX elemental map for the precursor graphite is showed. The EDX maps were acquired from random areas and additional spectra were obtained from areas with no carbon support. A more representative elemental analysis can come from SEM/EDX obtained over a dried drop of a concentrated suspension (Fig. 4c) deposited on silicon wafer. The results of this characterization are reported in Table 1. It is possible to see the very low and almost equal O/C ratios obtained. These results are coherent with the ones obtained by XPS analysis reported in Table 1.

**Fig. 5:**
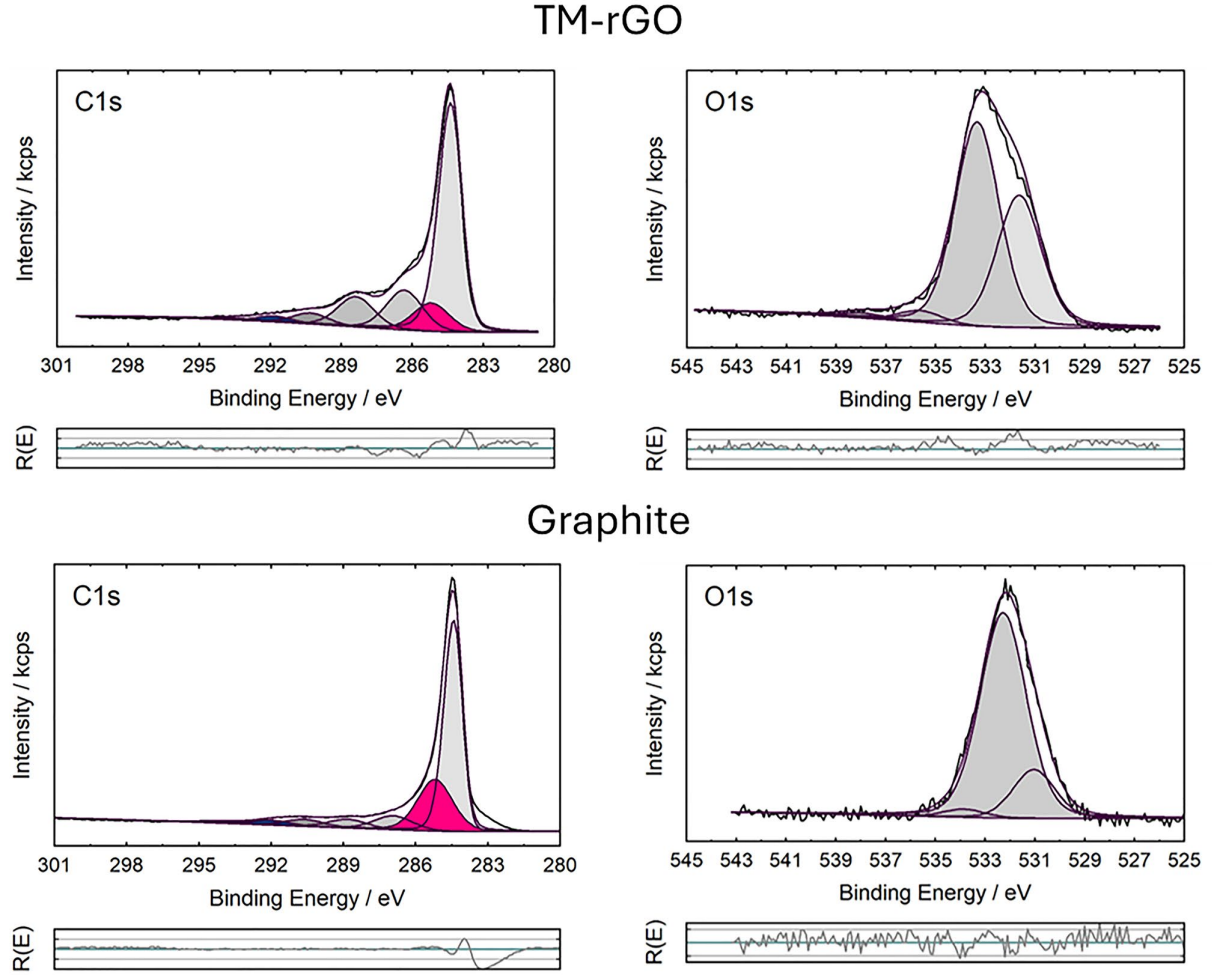
XPS characterisations of the material TM-rGO and of the graphite.

The O/C ratio obtained with XPS (see Fig. 5) is slightly higher than the ones observed with EDX. For the graphite reference sample, a much lower amount of oxygen was observed (O/C = 0.07), but significantly higher than with EDX. This can be explained with the higher surface sensitivity of XPS. In former experiments it was shown that the oxygen is located mainly at the outermost surface of stacks of graphene layers or graphite-like structures^34^. For more detailed insight into the surface chemistry of the materials high-resolution spectra were measured with XPS. As expected, the slightly asymmetric peak at 284.5 eV typical for sp^2^-hybridized carbon is the major component^35^. Furthermore, the π*-π satellite peaks around 292 eV were observed which are typical for the aromatic systems. Additionally, a component around 285 eV was observed which can be assigned to sp^3^-hybridized C-atoms bonded with other carbon or hydrogen. This is a hint for adventitious carbon which is always found with XPS^35^ or sp^3^ defect sites^36^. As expected, the percentage of C1s peaks with binding energies between 286 eV and 291 eV correlated with oxidized C atoms was higher for the TM-rGO material than for graphite. This agrees with the quantitative results described in Table 1. Alcohol and oxo-groups were identified from the C1s and O1s spectra. The percentage of the oxo-groups (E_B_= 531.5 eV) seems to be higher for TM-rGO than for the graphite precursor. The other main component are alcohol groups (E_B_= 533 eV)^37^.

**Table 1:** O/C ratios obtained with three different techniques for the synthetized TM-rGO and the graphite precursor.

Name	O/C_TEM/EDX_ (atom-% ratio)	O/C_SEM/EDX_ (intensity ratio)	O/C_XPS_ (atom-% ratio)
Graphite	> 99at% C	> 99at% C	0.07 ± 0.02
TM-rGO	0.21 ± 0.07	0.14 ± 0.00	0.28 ± 0.04

**Fig. 6:**
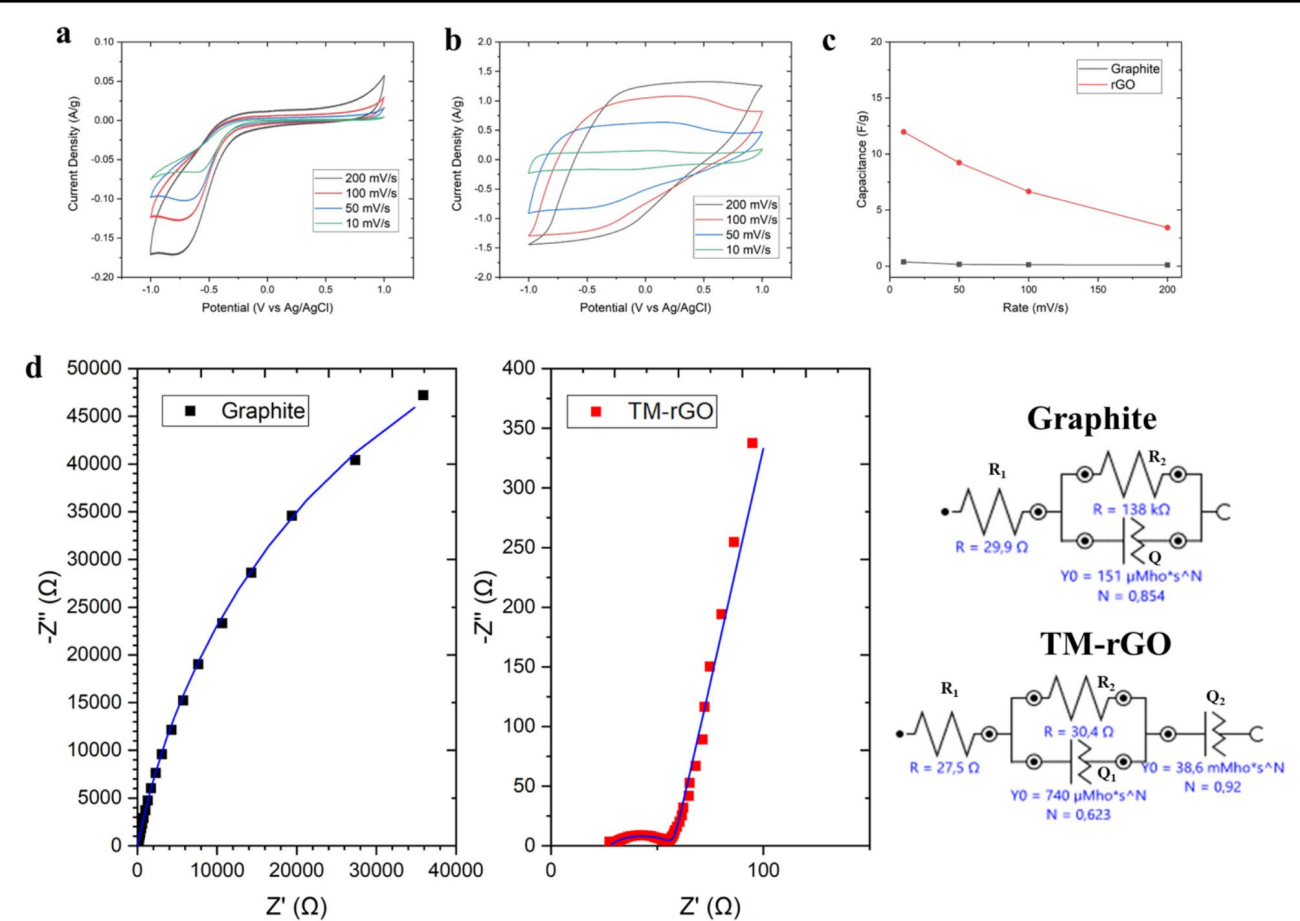
Cyclic Voltammetry at different scan rates for graphite (**a**) and TM-rGO (**b**). Comparison of the specific capacitances calculated from the CVs for both materials (**c**). Nyquist plots and equivalent circuits from EIS characterisation (**d**).

Graphene-based materials are usually employed in electronic devices due to its peculiar electrochemical characteristics^8^. Hence, additional to the compositional analysis above, we performed electrochemical characterization for graphite and TM-rGO (Fig. 6). For both the materials the current density grows with the scan rate, however, the growth is more significant for the TM-rGO (Fig. 6a, b,c). Capacitances calculated from the CVs show higher values at low scan rates as expected (Fig. 6c), as at low scan rates charges have more time to be transferred from the materials to the electrolyte. TM-rGO has specific capacitance of 3 to 12 F/g, which are nearly 30 times larger than those corresponding to graphite. The ability of TM-rGO to better store charge confirms the hypothesis that the original graphite was delaminated during the synthesis process. However, the treatment was not able to fully oxidize the material. This is because TM-rGO can reversibly store positive and negative charges, without irreversible redox reactions, and, thanks to its larger specific surface area compared with graphite, its capacitance increases accordingly.

The materials were also characterized by Electrochemical Impedance Spectroscopy (EIS) for evaluating the resistance to the charge transfer and the capacitance of the materials. The Nyquist plot (Fig. 6d) of the graphite electrode presented a single semicircle, characterized by impedance values above 40 kΩ. The data can be fitted very well with an R_1_(R_2_Q) equivalent circuit (Fig. 6d), where R_1_ is the uncompensated resistance due, for example, to the electrolyte, and R_2_ and Q are charge transfer resistance and capacitance of the graphite working electrode. The capacitance Q has been modelled as a constant phase element, to account for non-ideal capacitive behaviour of the real working electrode. Conversely TM-rGO showed a semicircle at high frequency with impedance below 50 Ω, and capacitive behaviour at low frequency. The data can be fitted very well with an R_1_(R_2_Q_1_)Q_2_ equivalent circuit, where R_1_, R_2_ and Q_1_ have the same meaning as for the graphite electrode. Q_2_ is an additional capacitance that must be added to fit the impedance at low frequency values. R_2_ is 138 kΩ for graphite and is reduced by almost four orders of magnitude to 30 Ω for TM-rGO. The values of TM-rGO Q_1_ and graphite Q are very similar, nevertheless TM-rGO Q_2_ is approximately two orders of magnitude larger, and its larger value can account for the larger capacitance observed for TM-rGO with CV. CV is considered a DC technique, even if the potential is cycled in the timescale of tens of seconds, and therefore CV studies the electrodes at their low frequency limit.

These results highlight the lower charge transfer resistance and larger capacitance for TM-rGO, compared with graphite, in accordance with the synthesis of a rGO. Moreover, the linear behaviour observed for TM-rGO in the Nyquist plot at frequencies between 0.01 and 1 Hz is again coherent with the synthesis of rGO, instead of graphene oxide. For graphene oxide materials a second semicircle in that frequency region, is observed instead, and it has been associated to a finite-length diffusion layer, whereas for rGOthe diffusion layer is semi-infinite, and no curvature is observed in the Nyquist plot between 10 Hz and 10 mHz^38^.

**Fig. 7:**
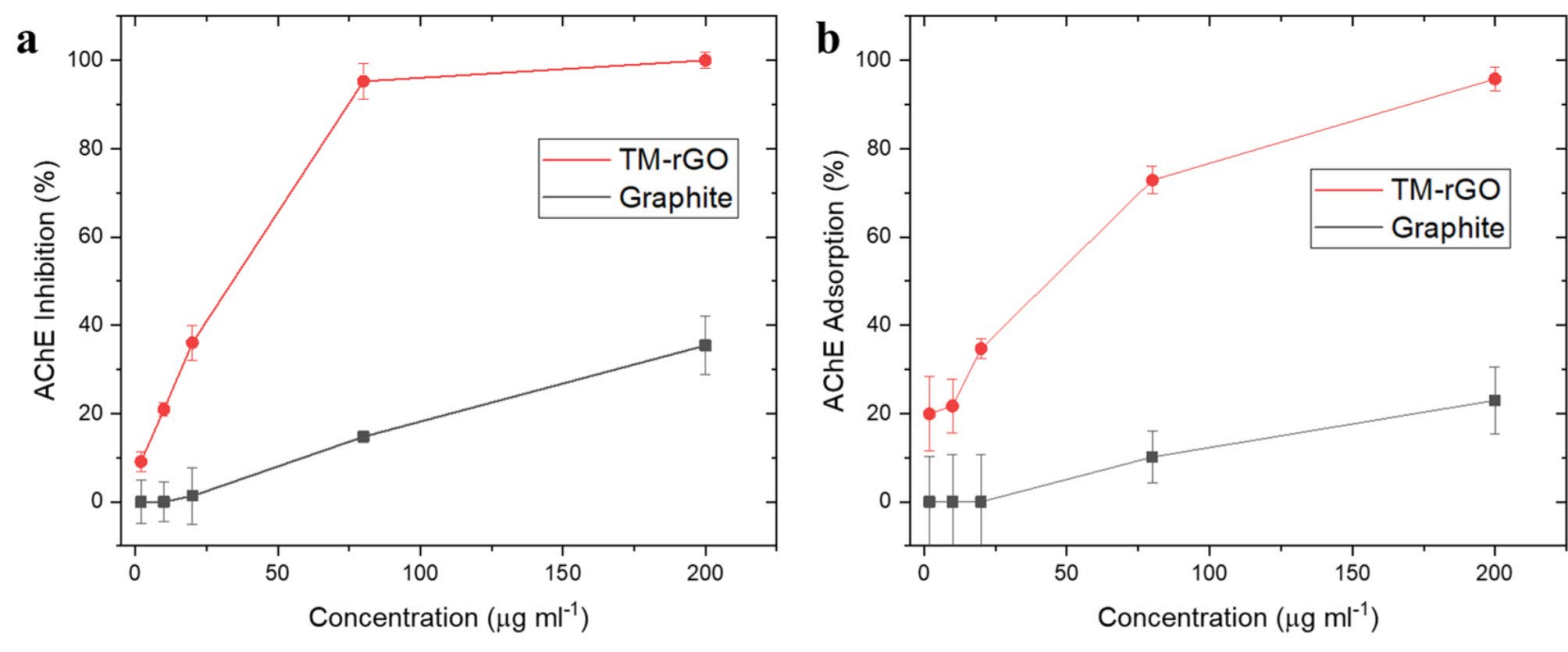
AChE activity inhibition (**a**) and adsorption (**b**) curves of the material TM-rGO (red) and of the original graphite (black).

Finally, the biological reactivity of the synthetized material and of the graphite was assessed by acetylcholinesterase enzyme (AChE) activity inhibition and adsorption^39^. Analyses of enzyme activity after their incubation with materials reflect differences in characteristics of tested materials. In Fig. 7 we report the inhibition and absorption curves of the TM-rGO and of the graphite. It is possible to see the great difference between the two materials, which could be due to the increase in TM-rGO specific surface area because of the exfoliation that occurred during the treatment.

This is also confirmed by higher adsorption of enzyme to TM-rGO compared to graphite. Adsorption of enzyme to material surface affects the structure of enzyme and subsequently its activity. Indeed, the inhibition of enzyme after incubation with TM-rGO is almost complete at 80 µg ml^−1^ while at the same concentration of graphite, the inhibition is lower than 20%. This confirms significant differences in characteristics of the two materials.

## Conclusions

For the first time, a One-Pot synthesis for rGO was developed. With an appropriate choice of the synthesis conditions of the Tour’s method, we obtained a material with very limited oxidation and characteristics that are more like rGO rather than the graphene oxide, as proven by different characterization techniques. This could be due to the combination of different synthetic parameters here used, the 50:50 use of H_3_PO_4_ and H_2_SO_4_ helps the intercalation of sulphates but it could be detrimental for the oxidation of the graphite. Moreover, we use a very poor amount of KMnO_4_ and, finally, the mixture was kept at 75 °C degree for 96 h, favouring a sort of direct thermal reduction of the material. Moreover, although a poor oxidation, we were able to obtain a good exfoliation at the end of the treatment. Therefore, we avoid any further sonication process that could damage the material increasing the presence of defects. The repeatability of the procedure was assessed replicating the synthesis, consistently achieving similar results. This new procedure can be exploited for directly synthesizing rGO in water without the use of reducing agents, allowing to reduce the toxicity and the costs of the reaction.

## Electronic supplementary material

Below is the link to the electronic supplementary material.

Supplementary Material 1

## Data Availability

All data generated or analysed during this study are included in this published article and its supplementary information files.
