# Publisher Correction: Development of a one-pot synthesis of rGO in water by optimizing Tour’s method parameters

**DOI:** 10.1038/s41598-024-77468-6

**Published:** 2024-11-19

**Authors:** Andrea Rossi, Eugenio Alladio, Damjana Drobne, Vasile-Dan Hodoroaba, Kerstin Jurkschat, Veno Kononenko, Loay Akmal Madbouly, Paul Mrkwitschka, Sara Novak, Jörg Radnik, Špela Saje, Rosangela Santalucia, Fabrizio Sordello, Francesco Pellegrino

**Affiliations:** 1 Department of Chemistry and NIS Centre, University of Torino, Via Giuria 7, 10125 Torino, Italy; 2 Department of Biology, Biotechnical Faculty, University of Ljubljana, Večna Pot 111, 1000 Ljubljana, Slovenia; 3 Federal Institute for Materials Research and Testing (BAM), Unter Den Eichen 44-46, 12203 Berlin, Germany; 4 Department of Materials, Oxford University, Begbroke Science Park, Begbroke Hill, Yarnton, Oxford, OX5 1PF UK

Correction to: *Scientific Reports* 10.1038/s41598-024-73606-2, published online 27 September 2024

The Acknowledgements section in the original version of this Article was incomplete.

“Ms. Thorid Lange (BAM) is acknowledged for the SEM and EDX measurements and Mr. Jörg-Manfred Stockmann (BAM) for the XPS analysis.”

now reads:

“This study has received funding from the European Union’s Horizon Framework under grant agreement No 101092796 (project ACCORDs). Funded by the European Union. Views and opinions expressed are however those of the author(s) only and do not necessarily reflect those of the European Union or European health and Digital Executive Agency (HADEA). Neither the European Union nor the granting authority can be held responsible for them. A.R., E.A., R.S., F.S. and F.P. also acknowledge support from the Project CH4.0 under the MUR program “Dipartimenti di Eccellenza 6 2023–2027” (CUP: D13C22003520001). Ms. Thorid Lange (BAM) is acknowledged for the SEM and EDX measurements and Mr. Jörg-Manfred Stockmann (BAM) for the XPS analysis.”

The original Article has been corrected.

