# Supplementary material for: Development of a one-pot synthesis of rGO in water by optimizing Tour’s method parameters
Source: Sci Rep. 2024 Sep 27;14:22381. doi: 10.1038/s41598-024-73606-2 (PMC11436994; doi:10.1038/s41598-024-73606-2)
Supplement: Supplementary file 1 — Supplementary Material 1 [file 41598_2024_73606_MOESM1_ESM.docx]

**Development of a One-Pot Synthesis of rGO in Water by Optimizing Tour's Method Parameters**

Andrea Rossi,^1^ Eugenio Alladio,^1^ Damjana Drobne,^2^ Vasile-Dan Hodoroaba,^3^ Kerstin Jurkschat,^4^ Veno Kononenko,^2^ Loay Akmal Madbouly,^3^ Paul Mrkwitschka,^3^ Sara Novak,^2^ Jörg Radnik,^3^ Špela Saje, ^2^ Rosangela Santalucia,^1^ Fabrizio Sordello,^1^ Francesco Pellegrino^1^*

^1^ Department of Chemistry and NIS Centre, University of Torino, via Giuria 7, 10125 Torino, Italy

^2^ Department of Biology, Biotechnical Faculty, University of Ljubljana, Večna Pot 111, SI-1000, Ljubljana, Slovenia

^3^ Federal Institute for Materials Research and Testing (BAM), Unter den Eichen 44-46, 12203 Berlin, Germany

^4^ Department of Materials, Oxford University, Begbroke Science Park, Begbroke Hill, Yarnton, Oxford, OX5 1PF, United Kingdom

**Materials**

Graphite powder, <20 μm, synthetic, Sigma Aldrich (CAS 7782-42-5); Potassium Permanganate, KMnO_4_, (≧ 99.0%) Sigma Aldrich (CAS 7762-64-7); Sulphuric Acid, H_2_SO_4_, (95.0-98.0%) Sigma Aldrich (CAS 7664-93-9); Orthophosphoric Acid, H_3_PO_4_, (≧ 85% wt. in H_2_O) Sigma Aldrich (CAS 7664-38-2); Hydrogen Peroxide H_2_O_2_, (35 % w/w in H_2_O), SAFC (CAS 7722-84-1). All the compounds were used as received without any further purification step. All aqueous solutions were prepared with ultrapure water Millipore Milli-Q™ (TOC <2 ppb, conductivity ≥18 MΩ cm).

**UV-Vis Characterization**

Extinction measurements in the UV–vis range were carried out with a Varian Cary “100 Scan” spectrophotometer. The extinction of the catalysts was measured on freshly prepared suspensions (20 mg L^−1^) and 1 cm optical path length. The suspensions were stable during the time scales necessary for the measurements, and the results of repeated measures were reproducible. Suspensions were not stirred during the measures. Likewise, they were not sonicated. UV-Vs spectra of the replicas show the same trend of the material TM-rGO.

**XRD Diffraction**

X-ray diffraction (XRD) patterns of the powders were recorded with an Analytical X’Pert Pro equipped with an X’Celerator detector powder diffractometer using Cu K_α_ radiation generated at 45 kV and 40 mA. The 2θ range was from 10° to 90° with a step size (°2θ) of 0.01 and a counting time of 0.6 s. Diffraction patterns of the replicas show the same trend of the material TM-rGO, suggesting the direct transformation to rGO.


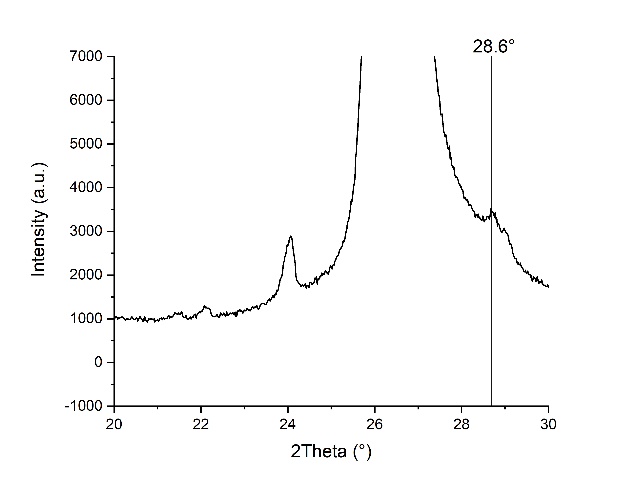


Figure S1. Focus on the 20-30° 2θ range for the graphite for highlighting the presence of impurity at 28.6°.

**Electron Microscopy**

The SEM/EDX characterization has been performed on all the samples after the preparation by drop-casting deposition of 6 µL liquid suspension on silicon substrates of 10 mm x 10 mm. The quite high concentration of all the suspensions (> 5 g/L) has resulted in a thick (several µm), mostly compact layer of material after drying in air for ~24 hours before introducing them into the high-vacuum chamber for SEM/EDX analysis, see Figure 4c for an overview of the sample rGO after its drop-cast deposition from the liquid suspensions in the as-received concentrations. Such a thick-coverage deposition is favourable for a microbeam analysis with EDX, while it is not convenient for a detailed SEM evaluation of the morphological parameters of the individual component structures of the selected samples. The EDX analysis was carried out at 5 kV. Graphite as the starting material has been also analysed by SEM/EDX, see Figure S4 with the expected result that only one main peak (of carbon) is present and very minor peak of oxygen, indicating a composition of >99% carbon.


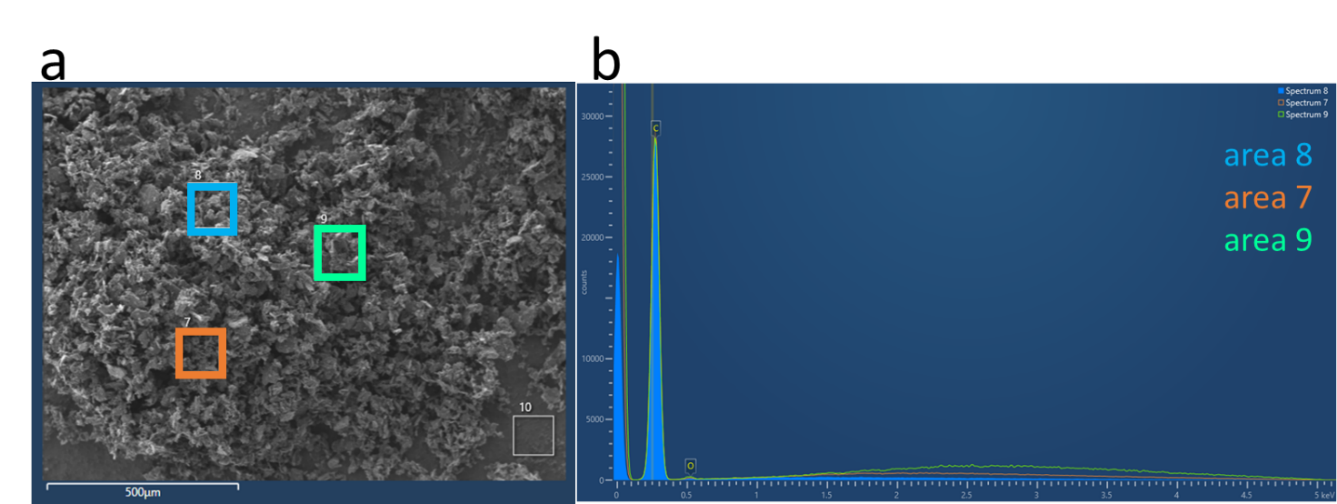


Figure S2. (a) SEM of graphite as the starting material with 3 marked areas for b) EDX analysis. Spectra are normalised to the height of the C peak in the EDS spectrum taken from area 8. The different Bremsstrahlung backgrounds are due to the different sample thickness at the respective area.

TEM samples were prepared by drop casting 10 µL of the dispersion (as received) onto 400 mesh holey carbon coated copper grids (AgarScientific) and left to dry for at least 1 hour at RT prior to TEM examination. Images and diffraction patterns were obtained on a 200 kV analytical JEOL 2100 instrument equipped with an Oxford instrument 80 mm thin window EDS detector and a Gatan Orius CCD camera. Image acquisition and processing was carried out using Digital Micrograph (GATAN version 3.51). In addition, some specific measurements were carried out on FIJI/ImageJ (version 1.54f). EDX spectra and maps were acquired and processed using Oxford Instruments Aztec software (version 5.1).

For a qualitative description of morphology and size several random images were taken at low and medium magnifications. Additional diffraction patterns were used to gather information on the extent of single or multi-layer crystalline areas. The particle size or more specifically the lateral dimensions of the graphite or graphene flakes were mostly too big for TEM measurements. The absolute upper limit for particle size measurement in TEM is limited by the size of the TEM grid square (approx. 30µm), realistically only particles that fall fully within a grid can be measured accurately and the upper size limit is approximately 20 µm. Overlapping particles as well as the irregular shapes make particle size estimates from TEM images unreliable. Drying platelets or sheet like samples onto TEM grids may result in additional changes in perceived shapes.

TEM-EDX maps were acquired from random areas and additional spectra were calculated from areas with no carbon support. Between 10 and 55 spectra were analysed for each sample and spectra where either oxygen or carbon had zero counts were excluded from the analysis. The amount of all additional elements detected was below 2at% in all areas and below 1% in the majority of cases.

*Graphite*

The starting material shows a variation of different shapes and morphology with graphite microplates and graphene/small layer graphite sheets in the hundreds of nanometres to more than 20µm size range (Figure S5). The graphitic micro plates tend to have straight edges, whereas the graphene like sheets often appear to display a wrinkled morphology with rounded edges. In many areas multiwalled graphitic structures in the size range 20-200 nm can also be detected. Graphite microplates show strong, well separated single reflections in the diffraction patterns indicating an extended range of single crystalline material. The diffraction patterns of the graphene like areas show an overlap of many single crystal patterns, sometimes almost merging into continuous diffraction rings due to many different overlapping orientations. EDX analysis showed that most areas show more than 99at% carbon, approximately 0.5at% oxygen and small amounts (< 0.2at%) of silicon (see Figure 4b).


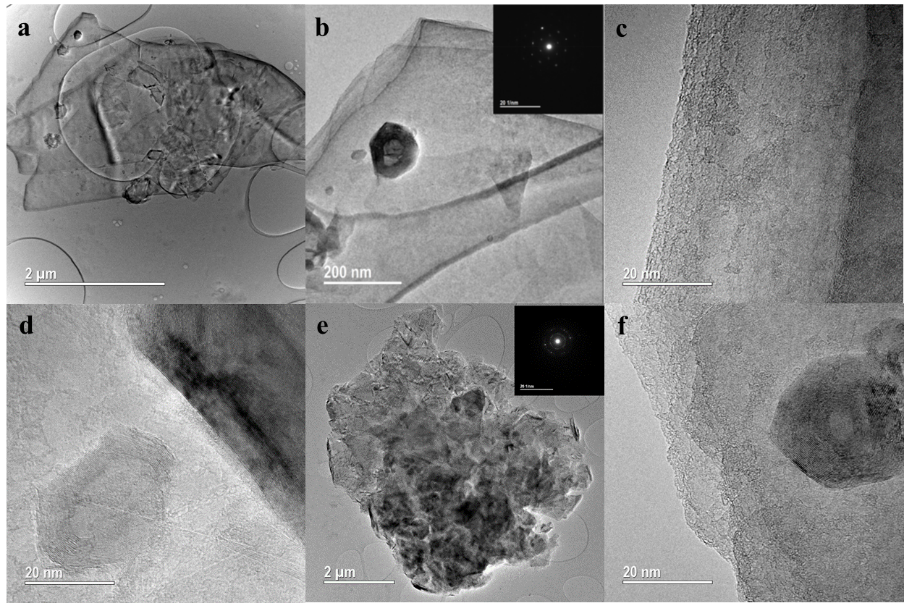


Figure S3. (a) Graphite microplate on holey carbon support film with a small number of layers, some multiwalled structures and mostly straight edges; (b) detail from area in (a) with the diffraction pattern taken from the flat multilayer area showing sixfold symmetry of overlapping areas; (c) HRTEM image from the edge of a microplate; (d) HRTEM micrograph of a multiwalled structure; (e) micrometre sized particle with partly wrinkled morphology and mostly rounded edges. The diffraction pattern shows almost ring like appearance due to a multitude of orientations of the individual graphene layers; (f) HRTEM micrograph from area in (e) with multiwalled structure and rounded edges.

*rGO*

Compared to the graphite powder starting material, the rGO shows a qualitative change in morphology with an increased portion of particles of single or small number of layers. Strong diffraction patterns with individual spots as well as darker BF contrast indicate graphite/graphite oxide of multiple layers, additional reflexes point to overlapping flakes of graphene or slightly oxidized graphene. A weaker BF contrast and rotationally extended DP reflexes indicate a small number of overlapping graphene layers partially oxidised and increased amount of oxidisation of these areas. In some areas the diffraction patterns show almost continues rings due to the multitude of orientations.

**Atomic Force Microscopy (AFM)**

Topology was investigated with atomic force microscopy (AFM) with a Park System XE–100 microscope in non-contact mode (using 910M ACTA cantilever). The samples were diluted in water to reach a concentration of 1 g/L and then deposited on a silicon wafer.

Topologies of the replicas were also investigated by AFM showing high reproducibility level. The replicas show the presence of a multilayer structure, with number of layers from 6 to 9.

**XPS Analysis**

The samples were prepared in the same ways as previously described for the SEM/EDX analysis. The Si wafer with the deposits were fixed on a sample holder with a double adhesive tape. The measurements were performed at an ULVAC-PHI “Quantes” spectrometer (Chanhassen, USA). For the measurements a monochromatic Al Kα source with an energy of 1486.6 eV was used. The spot size of the X-ray beam can be adjusted and was set to 100 µm for this study. The measurements were performed at three different areas of the samples. Photoelectrons were collected at an emission angle of 45°. The X-ray source is suited normal to the sample. The pressure in the sample chamber was kept below 10^-6^ Pa during the measurements. Low energy electrons and Ar^+^ ions were used for charge neutralization.

The survey spectra were performed with a step size of 1 eV at a pass energy of 280 eV and time per step of 200 ms. The measurements were repeated with 3 sweeps at an X-ray power of 25 W at 15 kV. The high-resolution spectra were measured with a pass energy of 54 eV and a step size of 0.1 eV at the same X-ray conditions.

The quantification was performed with the PHI MultiPak Software Version 9.9.1 using empirical sensitivity factors provided by the manufacturer. The binding energy scale was calibrated following a PHI procedure that uses ISO 15472 binding energy data.[1] The intensity was calibrated with the PHI MultiPak Software by a procedure introduced by Seah.[2] The peak fitting was performed with Unifit 2024 with sum Gaussian-Lorentzian curves and a modified Toogaard background.[3, 4].

*Table S1: XPS Fitting parameters*

| ***rGO*** | | | | | |
| --- | --- | --- | --- | --- | --- |
| *Peak name* | *Binding Energy /eV* | *LG-Mixing* | *FWHM/ eV* | *Asymmetry* | *relative peak area / %* |
| *C1s* | | | | | |
| *C=C (sp2)* | *284.5* | *0.1* | *1.1* | *- 0.05* | *47.2* |
| *C-C,C-H (sp3)* | *285.4* | *0.2* | *1.9* | *0* | *22.3* |
| ***C****-OH* | *286.7* | *0.2* | *1.9* | *0* | *10.9* |
| ***C****=O* | *288.4* | *0.2* | *1.9* | *0* | *10.8* |
| *O****C****=O* | *290.3* | *0.2* | *1.9* | *0* | *5.0* |
| *π-π** | *292.0* | *0.2* | *1.9* | *0* | *2.6* |
| *π-π** | *293.5* | *0.2* | *1.9* | *0* | *1.2* |
| *O1s* | | | | | |
| *C=****O*** | *531.6* | *0.2* | *2.1* | *0* | *35.3* |
| *C-****O****H* | *533.4* | *0.2* | *2.0* | *0* | *61.0* |
|  | *535.5* | *0.2* | *2.0* | *0* | *3.7* |

| ***Graphite*** | | | | | |
| --- | --- | --- | --- | --- | --- |
| *Peak name* | *Binding Energy /eV* | *LG-Mixing* | *FWHM/ eV* | *Asymmetry* | *relative peak area / %* |
| *C1s* | | | | | |
| *C=C (sp^2^)* | *284.4* | *0.1* | *0.9* | *- 0.05* | *54.4* |
| *C-C,C-H (sp^3^)* | *285.2* | *0.2* | *1.7* | *0* | *25.5* |
| ***C****-OH* | *286.9* | *0.2* | *1.9* | *0* | *7.7* |
| ***C****=O* | *288.8* | *0.2* | *1.9* | *0* | *4.8* |
| *O****C****=O* | *290.6* | *0.2* | *1.9* | *0* | *3.9* |
| *π-π** | *292.0* | *0.2* | *1.9* | *0* | *2.7* |
| *π-π** | *294.0* | *0.2* | *1.9* | *0* | *0.9* |
| *O1s* | | | | | |
| *C=****O*** | *531.8* | *0.2* | *2.2* | *0* | *45,3* |
| *C-****O****H* | *533.5* | *0.2* | *2.1* | *0* | *48.6* |
|  | *536.0* | *0.2* | *2.3* | *0* | *6.3* |

*Chemical Analysis of the replicas*

| **Name** | **O/C _TEM/EDX_ (atom-% ratio)** | **O/C _SEM/EDX_ (intensity ratio)** | **O/C _XPS_ (atom-% ratio)** |
| --- | --- | --- | --- |
| 1^st^ replica | 0.12 ± 0.05 | 0.10 ± 0.01 | 0.24 ± 0.02 |
| 2^nd^ replica | 0.11 ± 0.04 | 0.12 ± 0.02 | 0.25 ± 0.03 |

The O/C ratios from TEM-EDX analysis seem to be lower than the one reported for TM-rGO, however it must be underlined that the uncertainty for this kind of measurements is higher in comparison to the SEM-EDX and XPS (as underlined by the error range reported). This could be due to the presence of a carbon support (even if we tried to avoid the carbon for our measurements) and/or to a not perfect homogeneity of the samples.

**Raman Spectroscopy**

Raman spectra on the pellets obtained from freeze-drying of the suspensions of graphene samples were acquired using a Horiba iHR-320 Raman spectrometer, connecter to an Olympus microscope. This system uses the 532 nm line (max power 50 mW) of a DPSS laser for excitation and has Stokes-Raman shifted spectral coverage of 70–6700 cm^-1^. We used a 50x microscopic objective (NA = 0.50) to focus the laser beam (5 µm in diameter) onto the sample and to collect the Raman photons produced by the sample in back-scattering configuration.

Raman spectra of the replicas show the same trend of the material TM-rGO, with high defectivity and suggesting the presence of a multilayer structure.

**Infrared Characterisation**

The identification of functional groups in the samples was conducted by Fourier-transform infrared spectroscopy (FTIR) using the conventional transmission technique in KBr-pellets. Small amounts of sample were mixed with KBr powder to prepare the pellets. FT-IR spectra were recorded on a Bruker EQUINOX 55 FT-IR spectrometer equipped with a MCT detector, in the 4000–600 cm^-1^ spectral range, with a resolution of 4 cm^-1^ and by accumulating 128 scans to attain a good signal-to-noise ratio.

Infrared spectra of the replicas show the same features of the material TM-rGO, showing low water affinity and characteristics closer rGO.

**Electrochemical Characterization**

The electrochemical characterization was carried out on electrodes produced by dropping 1.0 mL of the graphene/graphite suspensions over a fluorine doped tin oxide (FTO) glass and subsequent drying in air at room temperature. The area of the electrodes is nearly 6 cm^2^. The electrochemical measurements were performed using a standard photo-electrochemical setup, composed of a computer-controlled potentiostat, AUTOLAB PGSTAT12. The electrochemical cell was a conventional three-electrode cell with a 1 mm thick fused silica window. The counter and reference electrodes were a glassy carbon and an Ag/AgCl/KCl (3 M) electrode, respectively. Cyclic Voltammetry (CV) measurements were carried out under a N_2_ atmosphere (flux 200 mL min^−1^); the solution (0.1 M KOH) was purged with N_2_ for 15 min before each measurement in order to eliminate the residual O_2_ present in the solution. The experiments are carried out at neutral pH. Scan rates for the CV were: 0.2, 0.1, 0.05 and 0.01 V s^−1^. Electrochemical impedance spectroscopy (EIS) was performed with the same setup, under a N_2_ atmosphere and at 0 V vs Ag/AgCl. The frequency scan was carried out between 10 kHz and 10 mHz. CV and EIS characterizations performed over the replicas show the same trend of the material TM-rGO, with higher capacitance and lower charge transfer resistance in comparison to graphite.

**AChE Inhibition Tests**

Adsorption and inhibition of AChE by TM-rGO and graphite was assayed by Ellman’s method as described in Mesaric et al. in 2013.[5] The AChE hydrolyses the substrate to form a product that reduces the colour indicator DTNB (5,5'-dithiobis-(2-nitrobenzoic acid)), producing the yellow chromogenic product 5-thio-2-nitrobenzoate (TNB). To assess the rate of enzyme inhibition, AChE, dissolved in 100 mM phosphate, was mixed with materials’ suspensions in different final concentrations (0–200 µg/mL). After the 10 min incubation, Ellman’s reagent and the substrate acetylthiocholine chloride were added to each tube and the enzyme reaction was allowed to develop for 5 min. Then the tubes were centrifuged for 5 min at 12,000 RCF to separate material–enzyme complexes from the free enzyme. In supernatant, the absorbance at 405 nm was measured 20 min after the addition of the substrate and the Ellman’s reagent to the reaction mixture, using the Tecan Infinite F Nano+ microplate reader. To determine the adsorption of the enzyme, AChE and materials were mixed and incubated for 10 min, centrifuged for 5 min at 12,000 RCF and the supernatants containing supernatants containing non-adsorbed enzyme pipetted onto the microtiter plate. Ellman’s reagent and substrate were added to each well and the absorbance was read at 405 nm exactly 20 min after the addition of the substrate and the Ellman’s reagent to the reaction mixture. Inhibition and adsorption tests over the replicas show similar results to the TM-rGO.

**Bibliography**

1. 15472:2010, I., *Surface Chemical Analysis – X-ray photoelectron spectrometers-Calibration of energy scales*. 2010: Geneve.

2. Tougaard, S., *Practical guide to the use of backgrounds in quantitative XPS.* Journal of Vacuum Science & Technology A: Vacuum, Surfaces, and Films, 2021. **39**(1).

3. Hesse, R., P. Streubel, and R. Szargan, *Product or sum: comparative tests of Voigt, and product or sum of Gaussian and Lorentzian functions in the fitting of synthetic Voigt‐based X‐ray photoelectron spectra.* Surface and Interface Analysis, 2007. **39**(5): p. 381-391.

4. Hesse, R. and R. Denecke, *Improved Tougaard background calculation by introduction of fittable parameters for the inelastic electron scattering cross‐section in the peak fit of photoelectron spectra with UNIFIT 2011.* Surface and Interface Analysis, 2011. **43**(12): p. 1514-1526.

5. Mesarič, T., et al., *Effects of surface curvature and surface characteristics of carbon-based nanomaterials on the adsorption and activity of acetylcholinesterase.* Carbon, 2013. **62**: p. 222-232.
